# Country and regional variations in purchase prices for essential cancer medications

**DOI:** 10.1186/s12885-017-3553-5

**Published:** 2017-08-24

**Authors:** Raphael E. Cuomo, Robert L. Seidman, Tim K. Mackey

**Affiliations:** 1 Joint Doctoral Program in Global Public Health, University of California, San Diego – San Diego State University, San Diego, CA USA; 2 Global Health Policy Institute, San Diego, CA USA; 3 Division of Health Management and Policy, Graduate School of Public Health, San Diego State University, San Diego, CA USA; 4 Department of Anesthesiology, University of California, San Diego – School of Medicine, San Diego, CA USA; 5 Division of Global Public Health, University of California, San Diego – School of Medicine, San Diego, CA USA

**Keywords:** Neoplasms, Health policy, Pharmacoeconomics, Economics, Global health, Public health, Health services accessibility, Medicine, Policy

## Abstract

**Background:**

Accessibility to essential cancer medications in low- and middle-income countries is threatened by insufficient availability and affordability. The objective of this study is to characterize variation in transactional prices for essential cancer medications across geographies, medication type, and time.

**Methods:**

Drug purchase prices for 19 national and international buyers (representing 29 total countries) between 2010 and 2014 were obtained from Management Sciences for Health. Median values for drug pricing were computed, to address outliers in the data. For comparing purchase prices across geographic units, medications, and over time; Mann-Whitney *U* tests were used to compare two groups, Kruskal Wallis *H* tests were used to compare more than two groups, and linear regression was used to compare across continuous independent variables.

**Results:**

During the five-year data period examined, the median price paid for a package of essential cancer medication was $12.63. No significant differences in prices were found based on country-level wealth, country-level disease burden, drug formulation, or year when medication was purchased. Statistical tests found significant differences in prices paid across countries, regions, individual medications, and medication categories. Specifically, countries in the Africa region appeared to pay more for a package of essential cancer medication than countries in the Latin America region, and cancer medications tended to be more expensive than anti-infective medications and cardiovascular medications.

**Conclusions:**

Though preliminary, our study found evidence of variation in prices paid by health systems to acquire essential cancer medications. Primarily, variations in pricing based on geographic location and cancer medication type (including when comparing to essential medicines that treat cardiovascular and infectious diseases) indicate that these factors may impact availability, affordability and access to essential cancer drugs. These factors should be taken into consideration when countries assess formulary decisions, negotiate drug procurement terms, and when formulating health and cancer policy.

## Background

According to the World Health Organization (WHO), the “primary intent” of a health system is “to promote, restore or maintain health” [1]. For individuals with cancer, this goal is best served by efforts to increase tertiary prevention initiatives, which serve to reduce the progression or worsening of disease. While advances in the modern era have allowed for the development of treatments with comparatively improved efficacy [2], little has been done to quantify the complex modalities in the context of accessibility and affordability which can lead to inefficiency in the delivery of cancer treatment.

Cancer itself is among the biggest modern threats to individual and population health [3]. The global cancer burden has been steadily increasing through the 20th and 21st centuries [4], and will likely continue to increase in the foreseeable future [5]. Consequently, the demand for cancer medications will also intensify [6]. In an effort to help guide decisions regarding procurement and coverage of essential medicines, the WHO has formulated a policy mechanism, the Essential Medicines List (EML), which is a list of medicines defined as drugs that “satisfy the priority health care needs of the population” [7].

Medications listed on the EML are supposed to meet a standard for efficacy, safety, and cost-effectiveness [8]. The EML standard for cost-effectiveness, in particular, is meant to ensure that domestic health agencies seeking to acquire an essential medication by including it on their national formularies would be able to do so without unreasonable financial burden [9]. Given that the prevalence of cancer is rising more rapidly in economically developing areas of the world compared to high-income countries [4, 10], it is critically important that research be conducted to shed light on whether essential cancer medications can actually be acquired at reasonable prices.

This study will attempt to address this important concern by exploring: (1) if some countries or regions pay more for essential cancer medications than others; (2) if countries with higher income or cancer burden pay more or less for essential cancer medication; (3) whether some essential cancer medications are more expensive than others; and (4) whether essential cancer medication is more expensive than other therapeutic categories of essential medicines.

Characterizing the sources of financial variation in cancer drug pricing is valuable in the development of international policy as it may help to reduce the likelihood that price determinations result in barriers to acquire essential cancer medicine for at-risk populations [11]. Furthermore, this information may aid national governments in identifying and resolving issues in their pharmaceutical supply chains that impede procurement of essential cancer medications [12]. This information may also be helpful for understanding variations in availability of treatment options for different cancers [13, 14].

The ultimate goal of this research is to generate information that can help address suboptimal access to cancer treatment by characterizing affordability of essential cancer medications. Future policies that are informed by findings from this research may allow for improved international access to essential cancer medication or exploration of different policy mechanisms to enhance affordability, thereby improving the likelihood of survival for individuals with cancer who would otherwise be unable to access needed medication [11, 15, 16].

## Methods

### Overview

In order to achieve the goals of this study, we conducted a series of comparative analyses on *median* prices paid for essential cancer medications contained in a drug procurement dataset that includes 19 national and international buyers (representing 29 total countries) obtained from Management Sciences for Health (MSH) for the period 2010–2014. Specifically, we made comparisons between median prices of essential cancer medications listed on the WHO EML by geography, cancer medication type, and date/time of procurement. Specific comparisons examined in this study are summarized in Table 1.

**Table 1:** Comparisons tested in this study. For all tests, the dependent variable was medication price

Category of Analysis	Independent Variable	Test Performed	Number of Groups
Geographic	Buyers	Kruskal Wallis *H*	19
Geographic	Region*	Mann-Whitney *U*	2
Geographic	GDP (nominal)^†^	Linear Regression	N/A
Geographic	All-Cancer Incidence (per 100,000)^‡^	Linear Regression	N/A
Medication	Essential Cancer Medications	Kruskal Wallis *H*	43
Medication	Essential Medication Categories	Kruskal Wallis *H*	3
Medication	Medication Formulations	Mann-Whitney *U*	2
Medication	Pre/Post Generic Approval Date^§^	Mann-Whitney *U*	2
Longitudinal	Year	Kruskal Wallis *H*	5

*Obtained from the United Nations [20]

^†^Obtained from the International Monetary Fund [21]

^‡^Obtained from the International Agency for Research on Cancer [22]

^§^Obtained from DrugBank [26]

Data from the MSH database included prices paid to purchase a package of specific essential cancer medication. With the exception of the comparative analysis across essential medication categories (where prices were only available for 2014), all analyses were done on prices from 2010 to 2014, inclusive.

### Data

Data for drug prices were obtained from the *International Drug Price Indicator Guide* (hereafter “*Guide”)* published by MSH, an international nonprofit focused on building programs to facilitate the improvement and strengthening of health systems. Pricing data are kept in a database maintained by the MSH Center for Pharmaceutical Management, a non-profit unit of MSH. MSH works with the WHO to obtain drug prices for the *Guide*. For this study, MSH used a prior version (April 2015) of the WHO’s EML to determine which medications it included in the *Guide* [17]. Pricing data from the *Guide* has been previously analyzed in published, peer-reviewed pharmacoeconomic studies assessing the affordability of essential medicines [18, 19].

Pricing data in the *Guide* are currently available for various formulations of nearly all cancer drugs included on the 19th edition (adopted in 2015) of the EML and provides data from 1996 to 2014 for 16 suppliers and 34 buyers. A supplier is a drug-selling entity that maintains a warehouse and provides a wide range of products, often including cancer drugs. A buyer is typically an agency within an individual national government (i.e., ministries of health), although some international buyers exist which represent a group of countries [17]. There were two international buyers of essential cancer drugs included in this study: the Organization of Eastern Caribbean States Pharmaceutical Procurement Service (OECS) and the Central American Integration System (SICA). The OECS represents seven countries (Antigua and Barbuda, Dominica, Grenada, Montserrat, Saint Kitts and Nevis, Saint Lucia, and Saint Vincent and the Grenadines) and the SICA represents eight countries (Belize, Costa Rica, the Dominican Republic, El Salvador, Guatemala, Honduras, Nicaragua, and Panama). However, it should be noted that Costa Rica, the Dominican Republic, and Guatemala also purchase cancer drugs via their own national agencies.

The *Guide* states that buyer prices, unlike supplier prices, do not require adjustment for shipping charges. Furthermore, the country identity of the purchaser was only available for buyer prices, and not for supplier prices. For these reasons, buyer prices were used in this study’s analysis. Furthermore, this study primarily used median values for measures of center, as the *Guide* recommends the use of median values for analytical purposes due to the propensity for outliers among available medication pricing data [17].

### Comparative analyses

Three sets of comparative analyses were conducted. Data management and statistical testing was conducted in R version 3.2.3 (R Foundation for Statistical Computing: Vienna, Austria), and graphs were produced using JMP version 10 (SAS Institute: Cary, North Carolina).

### Comparisons across geographies

First, the study sought to understand how the prices paid for essential cancer medications varied across different purchasing countries. We quantified the median price paid for all essential cancer medications for each buyer providing data. A Kruskal Wallis *H* test was conducted to test for a statistically significant difference in pricing data at the country level. International buyers were included in this comparative analysis.

Second, we sought to understand how the prices paid for essential cancer medications varied in different regions. We used country-level prices to compute a median value for each region. Therefore, countries and international buyers were attributed to regions. For this analysis, the United Nations Regional Groups classification scheme was used to categorize countries by region [20]. A Mann-Whitney *U* test was computed to test the null hypothesis that there was no statistically significant difference in prices between the two regions that emerged.

Third, we sought to understand whether there was an association between the prices that countries paid for essential cancer medications and the nominal GDP of those countries. We used linear regression to test the association between median country-level prices and country-level nominal GDP (available from the International Monetary Fund [21]).

Finally, we sought to understand whether there was an association between the prices that countries paid for essential cancer medications and disease burden from cancer in those countries. We used linear regression to test the association between median country-level prices and country-level all-cancer incidence. The all-cancer incidence rates for this study were obtained from the International Agency for Research on Cancer’s (IARC) Global Cancer (GLOBOCAN) data from 2012 [22]. These data exclude incidence from non-melanoma skin cancer, given its disproportionate impact on cancer burden metrics [23]. Therefore, with the exception of melanoma incidence, the combined incidence for the entire set of cancers was used, as was the median price for the entire set of essential cancer drugs. While some essential cancer drugs are used to treat a variety of cancers, others are almost exclusively used for specific cancer indications. Conversely, while some cancers can be treated by a variety of essential cancer drugs, others are almost exclusively treated by a very specific drug regimen.

#### Comparisons across medications

In this stage of analysis, we first sought to understand the variation in prices paid for different essential cancer medications. We quantified the median price for each cancer medication and assessed the range, midpoint, and distribution of median prices. A Kruskal Wallis *H* test was conducted to test for a statistically significant differences in prices among different cancer EML medications.

Second, we sought to understand how the prices paid for essential cancer medications may differ from the prices paid for essential medications in other therapeutic classes (i.e., medications that treat diseases other than cancer). We quantified the median price of essential cancer medication and the median price of essential medication in other categories, and then conducted a Kruskal Wallis *H* test to test the null hypothesis that no differences existed among category prices. The other categories chosen for comparison were those for cardiovascular disease and infectious disease.

The cardiovascular medicines category was chosen so as to allow for comparison of essential cancer medication pricing with that of another non-communicable disease that shares a similar epidemiological prevalence across global regions [24]. Conversely, the infectious disease category was chosen so as to allow for comparison with a set of diseases that has a very different etiology and global epidemiology [25]. For each category, the set of essential medications with data in the MSH database was cross-referenced with the set of essential medications in the 19th EML to ensure that the medication was also categorized as “essential”, and for the purposes of determining the percentage of essential medications in each category with available pricing data.

We also sought to understand if prices paid for injectable essential cancer medications were different from prices paid for orally administered essential cancer medications. The medication formulation was specified for each transaction (e.g. “vial,” “tab-cap,” “ampule,” etc.) [17]. We used a Mann-Whitney *U* test to determine if the prices for injectable formulations were significantly different from the prices for oral formulations.

Finally, we sought to understand whether the prices paid for generic/biosimilar cancer drugs were significantly different than the prices paid for branded cancer drugs. Information regarding the dates of approval for generic versions of these drugs were obtained from DrugBank, an online bioinformatics and chemoinformatics resource [26]. For drugs transitioning to generic availability within the time frame, and when sample size permitted statistical analysis, Mann-Whitney *U* tests were used to compare the median price for packages sold during the generic/biosimilar approval date and the median price for packages sold after the generic/biosimilar approval date.

#### Comparison across time

We also sought to understand how prices paid for essential cancer medications varied over time. We quantified the median price for each essential cancer medication for each year with available data between 2010 and 2014. Regarding the distribution of the number of package prices obtained across different years, it was observed that this distribution exhibited a skewness of −0.158 and a kurtosis of −1.411. These statistics support the uniformity of the distribution, thereby indicating that different time periods did not have different levels of influence on the entire multi-year sample. A Kruskal Wallis *H* test was used to determine if statistically significant differences in price existed across years. This was important to determine if there were significant price fluctuations experienced by countries in purchasing essential cancer medication over a five year time period.

## Results

A total of 949 transactions for essential cancer medication were recorded and analyzed from 2010 to 2014. The median price paid for a package of essential cancer medication was $12.63, with the lowest recorded price $0.03 and the highest recorded price $5250.00. This indicates a high degree of variation in overall essential cancer medication pricing.

### Comparisons across geographies

Prices were obtained for 19 buyers representing a total of 29 different countries. Results from a Kruskal Wallis *H* test suggest statistically significant differences in prices paid by different countries (*χ* ^2^ = 148.330; *p* < 0.001). The median price paid for essential cancer medication by each country is available in Table 2. The highest median price was for transactions from Namibia (*n* = 119; *M* = $27.75) and the lowest median price was for transactions from Ghana (*n* = 5; *M* = $1.10). While it is plausible that comparisons between certain countries may be biased due to the specific cancer medications purchased, the statistically significant difference in pricing detected in this analysis is supported by specific examination of a smaller sample of medications that had insufficient transactional data for statistical testing. For example, the Dominican Republic paid $4.56, on average, for 1 vial of cyclophosphamide in 2014 whereas Peru paid $9.52 for the same amount of the same drug in the same year. Similarly, Costa Rica paid $2.00 for 100 tablets of dexamethasone in 2010 whereas Namibia paid $7.96 for the same amount of the same drug in the same year. Many additional examples of the pricing disparity between countries for essential cancer medication are available from raw data in the MSH *Guide* [17].

**Table 2:** Median package prices of cancer medication for all included buyers, with other select characteristics

Buyer	UN Region	Median Price	GDP (mil, nominal)	Cancer Incidence
Barbados	Latin America	$14.66	$4498	263.1
Bolivia	Latin America	$4.94	$33,983	143.9
Botswana	Africa	$18.40	$12,701	107.6
Costa Rica	Latin America	$21.40	$56,908	179.3
Dominican Republic	Latin America	$6.15	$71,433	153.4
DR Congo	Africa	$9.60	$41,207	107.8
Ghana	Africa	$1.10	$38,171	91.7
Guatemala	Latin America	$6.24	$68,142	130.4
Lesotho	Africa	$6.28	$1766	103
Namibia	Africa	$27.75	$11,210	82.7
OECS	Latin America	$9.00	-	-
Peru	Latin America	$7.89	$178,643	154.5
Rwanda	Africa	$11.45	$8490	135.8
Senegal	Africa	$1.14	$14,572	101.2
SICA	Latin America	$8.15	-	-
South Africa	Africa	$14.27	$266,213	187.1
Sudan	Africa	$2.36	$93,729	91.1
Tanzania	Africa	$3.19	$45,899	123.7
Uganda	Africa	$12.17	$24,995	169.7

While the assessment of prices across geographies allows for insight into potential pricing variation among essential cancer medications, low sample sizes did not allow sufficient statistical power for stratified hypothesis testing. While 949 prices were available from the MSH database in the five-year time period, the average geography provided only 50 prices, with the range being 2 prices (Lesotho, Senegal) to 200 prices (South Africa). As this limited set of prices was divided among 43 drugs, sufficient statistical power did not exist to control for other characteristics of a geography, such as the mix of medications purchased. Furthermore, there was a wide range of prices available per essential cancer medication examined in the five-year time period, with the range being 3 prices (trastuzumab, vinorelbine) to 62 prices (cyclophosphamide). As with geographic comparisons, the limited set of prices per drug did not provide enough data to control for potentially influential variables corresponding to the drug, such as the mix of package doses. Therefore, while our analyses generate the possibility that a given country may have purchased a given cancer medication at a significantly different price than another country, other explanations for the observed variation are also possible.

To partially address this limitation in data availability, a sub-analysis was carried out to more closely examine the variation in transactional prices among buyers for certain individual cancer medications. This sub-analysis was conducted on cyclophosphamide (a cytotoxic drug used since 1959 for treatment of both blood cancers and solid tumors), which included the greatest number of package prices in the dataset during the five-year period (*n* = 62). Additionally, cyclophosphamide was available as a generic during this entire time frame (i.e. generic versions have been available since 1999) [26]. Buyers with less than five transactions recorded were excluded, leaving four buyers (Barbados, Namibia, the OECS multinational buyer, and South Africa) with 46 transactions. Purchases of tablets were excluded, allowing for the remaining 31 transactions to be normalized to the price per vial. A Kruskall Wallis *H* test among these 31 transactions indicated a statistically significant difference in prices (*χ* ^2^ = 9.105; *p* = 0.028), with the median price being $3.85 per vial for the OECS (*n* = 5), $5.29 per vial for Barbados (*n* = 5), $9.46 per vial for Namibia (*n* = 12), and $10.00 per vial for South Africa (*n* = 8). Further attempts to normalize to vial strength were limited due to sample size, resulting in insufficient statistical power for further testing.

To further address this data limitation, we also examined pricing data across the different non-tablet formulations of cyclophosphamide (200-mg vials, 500-mg vials, and 1-g vials) and observed appreciable variation in mean transactional prices: $0.006 per milligram for the OECS (*n* = 5), $0.012 per milligram for Barbados (*n* = 5), $0.015 per milligram for South Africa (*n* = 8), and $0.126 per milligram for Namibia (*n* = 12). Furthermore, when assessing only the price paid for the 1-g formulation (which provided the greatest number of transactions compared to the other formulations), we continued to observe noteworthy variations in transactional price: $6.39 per 1-g vial for Barbados (*n* = 3), $11.17 per 1-g vial for Namibia (*n* = 4), $3.85 per 1-g vial for the OECS (*n* = 5), and $13.02 per 1-g vial for South Africa (*n* = 4).

In regards to regional variation, transactions were recorded from 11 countries in the UN African region and 8 buyers (6 countries and 2 international organizations) in the UN Latin America region. Results from a Mann-Whitney *U* test suggest that transactions from African buyers exhibited a significantly higher price than transactions from Latin American buyers (*z* = −3.468; *p* = 0.001). The median price paid for essential cancer medication for an African buyer was $15.03 (*n* = 530), while the median price in these transactions for a Latin American buyer was $10.89 (*n* = 419). The significant difference in prices paid for essential cancer medication between these two regions further underscores a potential for pricing and procurement variation based on a country’s geographic region and possibly its procurement relationships.

Linear regression found no statistically significant relationship between country-level GDP (nominal) and median prices paid for essential cancer medication (β = −4.778 × 10^−6^; *p* = 0.867; R^2^ = 0.002). Linear regression also found no statistically significant relationship between country-level all-cancer incidence and median prices paid for essential cancer medication (β = 0.040; *p* = 0.347; R^2^ = 0.059).

#### Comparisons across medications

Results from a Kruskal Wallis *H* test suggest statistically significant differences in prices paid for different medications (*χ* ^2^ = 514.493; *p* < 0.001). The median price paid for each essential cancer medication is available in Table 3. The lowest median price paid for an essential cancer medication was for allopurinol (*n* = 17; *M* = $6.40) and the highest median price paid for an essential cancer medication was for trastuzumab (*n* = 3; *M* = $1800.00). Rituximab (*n* = 12; *M* = $413.53), capecitabine (*n* = 22; *M* = $354.04), and tioguanine (*n* = 6; *M* = $102.83) also exhibited median purchase prices exceeding $100 per package. As these medications are intended to be considered essential for health systems, prices over $100 per package likely impacts access for patients and coverage of these products by national health systems. Though efforts have been taken by some national governments to ameliorate the high price of trastuzumab [27, 28], these efforts have been geographically limited [29], with generally insufficient options for residents of developing countries to gain access to this treatment. It is worth noting that trastuzumab is a treatment for HER2-positive breast cancer, a form of breast cancer diagnosed for several hundred thousand women each year [30].

**Table 3:** Cancer medications in the 19th EML with median purchase price and formulations available

Essential Cancer Drug	Median Price	Oral	Injectable
Allopurinol	$1.56	X	
All-Trans Retinoid Acid	*Not Available*	X	
Anastrazole	$6.40	X	
Asparaginase	$42.58		X
Azathioprine	$14.28	X	X
Bendamustine	*Not Available*		X
Bicalutamide	$17.61	X	
Bleomycin	$18.24		X
Calcium Folinate	$15.98	X	X
Capecitabine	$354.04	X	
Carboplatin	$26.49		X
Chlorambucil	$32.83	X	
Ciclosporin	$88.64	X	
Cisplatin	$8.47		X
Cyclophosphamide	$13.00	X	X
Cytarabine	$4.27		X
Dacarbazine	$14.50		X
Dactinomycin	$25.23		X
Daunorubicin	$6.08		X
Dexamethasone	$2.01		X
Docetaxel	$44.65		X
Doxorubicin	$7.53		X
Etoposide	$4.57	X	X
Filgrastim	$52.11		X
Fludarabine	$91.46	X	X
Fluorouracil	$1.86		X
Gemcitabine	$25.85		X
Hydrocortisone	$1.96		X
Hydroxycarbamide	$16.67	X	
Ifosfamide	$25.01		X
Imatinib	$75.41	X	
Irinotecan	*Not Available*		X
Leuprorelin	*Not Available*		X
Mercaptopurine	$55.90	X	
Mesna	$3.47	X	X
Methotrexate	$9.00	X	X
Methylprednisolone	$9.06		X
Oxaliplatin	$44.55		X
Paclitaxel	$14.85		X
Prednisolone	$7.18	X	
Procarbazine	*Not Available*	X	
Rituximab	$413.53		X
Tamoxifen	$5.24	X	
Tioguanine	$102.83	X	
Trastuzumab	$1800.00		X
Vinblastine	$14.75		X
Vincristine	$3.53		X
Vinorelbine	$18.10		X

Transactions were recorded for 43 of the 48 medications (90%) in the “Antineoplastic and Immunosuppressives” category of the 19th EML, 81 of the 167 medications (49%) in the “Anti-Infective Medicines” category, and 18 of the 28 medications (64%) in the “Cardiovascular Medicines” category. Results from a Kruskal Wallis *H* test suggest statistically significant differences in prices between categories (*χ* ^2^ = 108.421; *p* < 0.001). The median price paid for a package of essential cancer medication in 2014 was $9.31 (*n* = 204), which is approximately 4 times higher than the median price paid in 2014 for a package of essential infectious disease medication (*n* = 457; *M* = $2.45) and 5 times higher than the median price paid for a package of essential cardiovascular disease medication (*n* = 129; *M* = $1.73). This analysis indicates that the median prices for acquiring essential infectious disease medication and essential cardiovascular disease medication are much lower than the median price of acquiring essential cancer medication.

Results from a Mann-Whitney *U* test suggest that prices paid for injectable cancer medications were not significantly different than prices paid for oral cancer medications (*z* = −0.205; *p* = 0.837). The median price for a package of injectable medication was $12.17 (*n* = 610) and the median price for a package of oral medication was $13.31 (*n* = 339). About two-thirds (64.3%) of transactions were for injectable cancer medications.

We also investigated the role of generic/biosimilar availability on transactional prices of essential cancer drugs. Among the 43 drugs with available prices, 34 had generic versions available throughout the entire 5-year period from 2010 through 2014 (median price = $10.62), 3 had no generic versions throughout this entire period (median price = $29.35), and the remaining 6 had a generic approved during the 5-year period. Drugs that became available as generic are anastrozole (generic available in 2010), gemcitabine (2010), docetaxel (2012), capecitabine (2012), rituximab (2013), and imatinib (2013). Only docetaxel and gemcitabine had greater than 5 package prices available in each of the following two categories: (1) the years from 2010 up through the generic approval year and (2) the years after the generic approval year. The year of approval was included in the “before” category, as this would allow for a short period of time for international buyers to adapt to ordering from new drug manufacturers. When using Mann-Whitney *U* tests to compare prices of these drugs before and after generic versions were approved, we found that docetaxel exhibited a statistically significant decrease in median price from $53.84 to $27.89 (*z* = −2.134; *p* = 0.033) and capecitabine exhibited a non-significant decrease in median price from $362.84 to $209.68 (*z* = −1.280; *p* = 0.201).

#### Comparison across time

Results from a Kruskal Wallis *H* test suggest that, between 2010 and 2014, prices paid for essential cancer medications did not significantly differ by year (*χ* ^2^ = 3.497; *p* = 0.478). Indeed, prices did not appear to exhibit a clear longitudinal trend, with the median price for essential cancer medication being $12.41 in 2010 (*n* = 216), $14.90 in 2011 (*n* = 150), $14.77 in 2012 (*n* = 131), $12.81 in 2013 (*n* = 248), and $9.31 in 2014 (*n* = 204).

## Discussion

Though preliminary and limited to pricing and procurement data available from the MSH dataset, the results of this study suggest that: (1) some countries and regions may pay more for essential cancer medication than others; (2) some essential cancer medications are likely significantly more expensive than others; (3) essential cancer medications reviewed exhibit higher prices than certain other categories of essential medications; and (4) it is possible that prices for essential cancer medications may decrease after the introduction of a generic product.

Importantly, as a global policy-based mechanism, the EML has the potential to have a broadly permeating downstream effect, whereby national, subnational, and private-sector participants in the pharmaceutical supply chain react to EML medication inclusion by creating more market demand and potentially decreasing pricing due to increased purchasing volume that could lead to broader availability [31, 32]. However, the longitudinal analysis in this study did not uncover evidence that the EML has met its potential to have a beneficial effect on medication affordability or availability through evidence of decreased prices.

While it is already known that the geographic location of a patient will likely have a notable impact on the patient’s treatment options [33], and therefore the patient’s ability to survive [33], the specific mechanisms by which location influences odds of survival have not been comprehensively enumerated. In this study, we observed a variation in the prices paid by country-level organizations in acquiring important medication for their cancer patients, including in our sub-analysis of specific essential cancer drug medications. This indicates that geographic location may act as a potential mediator in pricing variation, given that different prices for the same drug are being paid by different health systems. This could have an impact on treatment availability, quality, and coverage, as it may lead to procurement failures of essential drugs or suboptimal formulary inclusion of medications with poorer treatment options [34].

The variation in regional and country-level drug pricing observed in this study also implies that current EML policies do not take into account geographic and therapeutic class characteristics that may directly impact procurement terms that translate to drug access and affordability. Other pharmaceutical policy mechanisms, such as tiered pricing, group purchasing organizations (to increase purchase power and negotiate lower pricing), and lowering tariffs may result in more targeted medication pricing sensitive to the geographic, economic, and population health characteristics unique to each country and help promote more equitable access [35]. Consequently, a fundamental ethical question raised is the appropriateness and equity of a country’s trade policy to impact drug procurement prices for one country but not another [36–38]. This same question is raised with regard to the impact of trade policies on the price of drugs for some disease classes but not drugs for other disease classes.

Overall, our preliminary findings indicate that geography and cancer medication type have a role to play in country and regional-level drug pricing. However, limitations of the data set reviewed (discussed below) and the need to account for additional factors associated with cancer drug pricing and procurement that could not be stratified, necessitate further study, analysis, and better data collection. Despite these limitations, as of this study date, the MSH dataset is the arguably the best publicly available dataset to conduct this preliminary examination. Hence, results from this study can help inform future studies and hypothesis-driven analysis on the association between essential medication classification, pricing, and the factors examined.

### Limitations

This study may not have comprehensively examined all independent variables which could explain variations in prices for essential cancer medication. It was also not possible to determine if data were gathered for the *Guide* in a non-random fashion. Furthermore, low sample sizes for the prices attributed to an individual drug did not permit for meaningful comparison of median geographic prices to be stratified by drug. Similarly, low sample sizes for individual buyers did not permit comparison of median drug prices to be stratified by buyer.

This study’s analysis involved covariates that were ecological and oftentimes aggregated, including measures such as all-cancer incidence and national GDP at nominal rates. While the ecological and aggregate nature of these covariates allow for hypothesis-generating observations at the macro level, more robust analysis to better confirm these associations would benefit from data derived at a more resolute level. With regard to aggregate measures of wealth, caution should be taken in determining whether pricing associations being studied pertain to the capacity of national health systems to procure cancer medications or the financial capacity of individual patients to obtain cancer medications, both critical factors in assessing access to and affordability of essential medicines.

Nonetheless, in order to support findings that showed significant differences in the price of agglomerated cancer drugs across buyers, this study provided a sub-analysis comparing median prices of the cancer drug with the highest sample size (cyclophosphamide) across buyers with the highest numbers of recorded transactions. Also, in order to minimize the potential impact of missing variable bias, we described the variation in cancer drug prices across a number of pharmacoeconomic factors. Furthermore, prices were gathered by the MSH via a systematic methodology, thereby theoretically providing a degree of control for sampling bias that would have otherwise differentially influenced buyers in the dataset.

The large range of chemotherapy prices observed in this study indicates that certain chemotherapies could be preferred over others in regional or country-level healthcare facilities. This may lead to suboptimal treatment or to restrictions on the number of patients for which expensive chemotherapies may be provided. Significant associations uncovered in this study should be considered hypothesis generating, and further studies should be conducted that compare prices paid for essential medication between geographies, cancer medication categories, and individual classes of drugs. Better transparency and greater data availability are necessary in order to conduct analyses that yield more conclusive findings and provide a better overall picture of affordability and access to essential cancer medications globally.

## Conclusions

Over one-fifth of essential chemotherapies assessed in this study cost over $50 per package. As over 50 countries have a per capita gross national income of under $2000 per person, essential chemotherapy may be too expensive to ensure adequate regional or country-level access. This study also found that countries with smaller economies were not being sold chemotherapy at lower prices than countries with larger economies. Global price barriers to chemotherapy access will likely be exacerbated in the future, as the number of cancer patients in low- and middle-income countries is expected to rapidly rise.

## Abbreviations

EML: WHO Essential Medicines List
GDP: Gross Domestic Product
GLOBOCAN: IARC Global Cancer Data Project
IARC: International Agency for Research on Cancer
MSH: Management Sciences for Health
OECS: The Organization of Eastern Caribbean States Pharmaceutical Procurement Service
SICA: The System of Central American Integration
WHO: World Health Organization
